# MiRNA profile in CD4 positive T cells from HTLV-2 and HIV-1 mono- and co-infected subjects

**DOI:** 10.1186/1742-4690-9-S1-P2

**Published:** 2012-05-25

**Authors:** E Pilotti, C Casoli, MV Bianchi, F Bignami, Francesca Prati

**Affiliations:** 1 GEMIB Laboratory, Parma, Italy; 2 Hospital Santa Maria Nuova, Reggio Emilia, Italy

## Introduction

The HTLV-2/HIV-1 co-infection has been shown to be associated with a delayed progression to AIDS (Turci et al. 2007). Casoli et al. (2007) have demonstrated the key role played by CCL3L1 chemokine, whose expression is induced by HTLV-2 infection, in slowing down HIV-1 disease. MiRNAs (miRNAs) are small non-coding RNAs that regulate fundamental cellular processes. Since HTLV-2 creates a cellular environment favourable to itself and adverse to HIV-1, it is supposable that host miRNAs profile can be modulated by HTLV-2 to this aim. Here, we investigated the expression profile of miRNAs in HTLV-2 and HIV-1 mono-infected, in double infected, and HIV-1 exposed uninfected (MEU) subjects.

## Materials and methods

CD4+ T cells were purified from blood samples of 7 LTNP HTLV-2/HIV-1 co-infected, 5 HIV-1^MEU^, 7 HTLV-2/HIV-1^MEU^, 10 viremic and 7 LTNP HIV-1 mono-infected individuals and 10 healthy donors as controls. The expression profile of 377 miRNAs was obtained by real-time quantitative PCR and 2^-ΔΔCt^ method, by which the PCR signal of miRNAs transcript in CD4+ T-lymphocytes from infected subjects were related to that of healthy donors. By real time PCR, each cohort of subjects was also tested for the expression of miRNA processing enzymes, Dicer and Drosha.

## Results

Analyzing these miRNA identified by the comparison of the two viral infection, 4 miRNAs (329, 337-5p, 379, and 503) were down-regulated and 6 miRNAs (34a, 125a-3p, 155, 203, 449a, and 502-5p) were up-regulated in both conditions, suggesting a retroviral exposure signature. HTLV-2/HIV-1^MEU^ subjects are characterized by a miRNA profile similar to that of healthy donors, while a strong up-regulation of miRNA profile marked subjects infected by o exposed to HIV-1. Dicer and Drosha expressions seem to explain the miRNAs changes observed.

## Conclusions

These findings enable us to better understand the potential role of miRNAs in the development of resistance to HIV-1 infection by HTLV-2.

